# Editorial: Current Advances in Soft Robotics: Best Papers From RoboSoft 2018

**DOI:** 10.3389/frobt.2020.00056

**Published:** 2020-04-21

**Authors:** S. M. Hadi Sadati, Perla Maiolino, Fumiya Iida, Thrishantha Nanayakkara, Helmut Hauser

**Affiliations:** ^1^Department of Surgical and Interventional Engineering, King's College London, London, United Kingdom; ^2^Department of Engineering Science, Oxford Robotics Institute, University of Oxford, Oxford, United Kingdom; ^3^Department of Engineering, University of Cambridge, Cambridge, United Kingdom; ^4^Dyson School of Design Engineering, Imperial College London, London, United Kingdom; ^5^Department of Engineering Mathematics, University of Bristol, Bristol, United Kingdom

**Keywords:** soft robot, soft actuator, soft sensor, continuum manipulator, fiber jamming, growing, 3D printing, learning-based modeling

The field of Soft Robotics extends the notion of conventional robotics by using material and actuation systems that go beyond rigid body parts and electric motors. Often, soft robots are inspired by soft and compliant structures in biological creatures. The result is a new, remarkable set of systems and solutions with high dexterity, reconfigurability, multi-functionality, and robustness. Furthermore, the Soft Robotics approach enables us to build mechanisms for growing and healing. Soft Robotics opens a new design space by developing and using novel materials and manufacturing approaches. The Soft Robotics community takes inspiration from nature and employs a highly multi-disciplinary approach involving several disciplines such as Material Science, Chemistry, Applied Mathematics, Physics, Biology, Control Theory, and Computer Science, etc. As a results, Soft Robotics can provide novel solutions for challenges that weren't possible or hard to overcome with conventional, rigid approaches. However, using soft technologies also poses new challenges, especially, related to sensing and actuation, modeling and control, and manufacturing and durability. Soft Robotics is a research field still in its infancy, but it has remarkable potential. Nevertheless, it still has to overcome a number of challenges before it can deliver off-the-shelf solutions. To address these challenges and form a community, the first IEEE international conference of Soft Robotics (RoboSoft 2018) was organized in Livorno (Italy) on 24-28 April 2018. This special issue includes 10 articles from this conference which have been picked by a committee. These papers highlight some of the challenges and new trends of the field.

For example, Soft Robotics is at the frontier of bio-inspiration. It uses solutions found by natural evolution as a source of inspiration. Along this lines, Del Dottore et al. proposed a kinematic model for the tip motion of growing robots to navigate 3D environments while negotiating confined spaces and large cavities by adapting their body. However, bio-inspiration can go even beyond simply copying biological systems. Chen et al. introduced RUBIC (the Rolling, Untethered, Ballooning, Intelligent Cube) uses controlled inflation to roll from one face of the cube to another, in any one of four planar directions. The result is a safe and predictable locomotion in complex environments. Another challenge is to design soft sensors and actuators. They must be flexible themselves. Furthermore, an integrated manufacturing approach is required to guarantee higher durability and repeatability of the system. Yirmibeşoğlu et al. showed the superior reliability of 3D printed pneumatic actuators with their molded counterpart made of polydimethylsiloxane (PDMS) when exposed to high radiation. For soft sensing, especially proprioceptive sensing capabilities are needed for precise motion and deformation control. The paper by Shih et al. presents a method which exploits 3D printing technology and commercial available 3D printing material to integrate resistive sensors in soft actuators. This provides a fully integrated system with proprioceptive capability and high repeatability. Soft actuation is another challenges. The field offers a wide range of possible approaches, like Dielectric Elastomer Actuators (DEA), Shape Memory alloy (SMA), and many other. For example, Minaminosono et al. use DEA to design a soft rotational motor that continues to function even under deformation. Cao et al. use DEAs to build a Micro Air Vehicle. It takes inspiration from insects that use their elastic thorax and muscle system as a damped oscillator to flap their wings at resonant frequency. The paper successfully demonstrated the use of DEA technology to implement such a design. While softness provides new capabilities, it also inherently carries new limitations. For example, compliant materials are often lacking durability, which is a key requirement for industrial systems. Dåmmer et al. proposed a Finite Element Approach framework for the optimized design of polyJet-printed bellows actuators and showed that a design made of Agilus30™soft material could withstand more load cycles, but suffer from material characteristic time dependency compared to one with TangoBlackPlus™material. Application-specific designs are another direct pathway for soft robots into real-world applications. Gong et al. presented an underwater robot equipped with a pneumatic manipulator benefiting from an opposite-bending-and-extension mechanism for delicate manipulation of irregularly shaped seafood animals of different sizes and stiffness at the bottom of the natural oceanic environment. Precision, often required in real-world scenarios such as surgical interventions, asks for high-stiffness modes in soft robot. Brancadoro et al. investigated fiber jamming transition as an effective technological approach for obtaining variable stiffness in slender soft structures. Finally, control challenges posed by soft robot composed of irregular shapes, and complex deformations are also one of the bottlenecks for industrial deployment. Learning-based control methods can offer flexibility and precision without a detailed theoretical model of the robot. Hyatt et al., for example, investigated model-based control of a pneumatic actuator using learned nonlinear discrete-time models and showing the potential of combining empirical modeling approaches with model-based control.

This special issue is a snapshot of current challenges in Soft Robotics. It highlights the great potential of the field, but also shows the number of exciting research questions it can offer. This includes the use of bio-inspiration, the development of novel soft actuation, sensing and computation capabilities, the integration of various materials with the help of novel manufacturing approaches, and the search for improved model and optimization frameworks, and many others. Using a highly interdisciplinary approach Soft Robotics has significantly extended the tool box for robot design and, therefore, enabled solutions to previously unsolved problems. We have now potential solutions at our fingertips for safe human-robot interaction, non-invasive surgery, robust autonomous locomotion, soft grippers for agricultural application, safe rehabilitation systems, haptic interfaces, and many others. The field is growing fast and soon Soft Robotics will be an integral part of a the common robotics approach.

## Author Contributions

All authors listed have made a substantial, direct and intellectual contribution to the work, and approved it for publication.

## Conflict of Interest

The authors declare that the research was conducted in the absence of any commercial or financial relationships that could be construed as a potential conflict of interest.

